# Size Control and Enhanced Stability of Silver Nanoparticles by Cyclic Poly(ethylene glycol)

**DOI:** 10.3390/polym14214535

**Published:** 2022-10-26

**Authors:** Yubo Wang, Jose Enrico Quijano Quinsaat, Feng Li, Takuya Isono, Kenji Tajima, Toshifumi Satoh, Shin-ichiro Sato, Takuya Yamamoto

**Affiliations:** 1 Graduate School of Chemical Sciences and Engineering, Hokkaido University, Sapporo 060-8628, Hokkaido, Japan; 2 Division of Applied Chemistry, Faculty of Engineering, Hokkaido University, Sapporo 060-8628, Hokkaido, Japan

**Keywords:** polymer topology, PEG, AgNPs, size control, stability

## Abstract

Silver nanoparticles (AgNPs) are used in a wide range of applications, and the size control and stability of the nanoparticles are crucial aspects in their applications. In the present study, cyclized poly(ethylene glycol) (*c*-PEG) with various molecular weights, along with linear PEG with hydroxy chain ends (HO–PEG–OH) and methoxy chain ends (MeO–PEG–OMe) were applied for the Tollens’ synthesis of AgNPs. The particle size was significantly affected by the topology and end groups of PEG. For example, the size determined by TEM was 40 ± 7 nm for HO–PEG_5k_–OH, 21 ± 4 nm for *c*-PEG_5k_, and 48 ± 9 nm for MeO–PEG_5k_–OMe when the molar ratio of PEG to AgNO_3_ (*ω*) was 44. The stability of AgNPs was also drastically improved by cyclization; the relative UV–Vis absorption intensity (*A/A*_0_ × 100%) at *λ*_max_ to determine the proportion of persisting AgNPs in an aqueous NaCl solution (37.5 mM) was 58% for HO–PEG_5k_–OH, 80% for *c*-PEG_5k_, and 40% for MeO–PEG_5k_–OMe, despite the fact that AgNPs with *c*-PEG_5k_ were much smaller than those with HO–PEG_5k_–OH and MeO–PEG_5k_–OMe.

## 1. Introduction

Metal nanoparticles have been widely used in catalysis [1], bio-sensors [2], optics [3], electronic components [4], and biomedical treatments [5] in recent years. Due to the surface plasmon resonance (SPR) interaction between light and the surface of metal nanoparticles, various intrinsic particle properties such as size and shape affect the color and dispersion statuses of the colloidal solution [6]. In order to preserve their properties, the stability of the nanoparticles is a crucial aspect which has to be taken into account. Generally, nanoparticles are kept in solution. since their agglomeration is facilitated in the dry state, due to the presence of capillary forces. However, the stability of nanoparticles in solution is often disturbed by a range of factors such as light [7], heat [8], salt [9], and pH [10], resulting in aggregation of nanoparticles or changes in the color of the colloidal solution. In order to contain these phenomena, small molecules or polymers are dispersed into the colloidal solution, which are then adsorbed onto the particle surface. While molecules such as sodium citrate [11] and cetyltriethylammonium bromide (CTAB) [12] can stabilize particles from agglomeration as a result of electrostatic repulsion through the presence of negative and positive charges, respectively, incorporated in their structures, neutral polymers such as poly(ethylene glycol) (PEG) and poly(vinyl pyrrolidone) (PVP) mainly create steric hindrance in order to protect the particle surface, which is reported to be more effective than the charge stabilization mechanism [13]. On the other hand, polymers equipped with charges in their backbone structure, such as poly(sodium acrylate), can combine both stabilization mechanisms [14].

In the last decade, PEG with a long alkoxy chain has received more attention from researchers, due to its water solubility and biocompatibility, which can provide steric hindrance for the preparation of gold nanoparticles (AuNPs) or silver nanoparticles (AgNPs) [15]. In relation to this, PEG coverage on the surface of nanostructures was reported to be crucial for the ligand exchange process [16], and for controlling the shape of nanocrystals [17]. In addition, PEG is also often used to disperse/stabilize AuNPs or AgNPs in immunoassays, thus maintaining the dispersion of the particles and avoiding tracking by the immune system [18]. In particular, the physiological environment is an important factor for in vivo and in vitro experiments, and increasing salt concentration or ionic strength can lead to the aggregation or dissolution of nanoparticles [19]. In general, the addition of NaCl to a metal nanoparticle solution, together with the presence of oxygen, leads to the catalyzed dissolution of the particles, attributed to the corrosive nature of the chloride ions [20]. Furthermore, Li et al. reported the bridging of individual particles by secondary precipitates of AgCl when AgNPs were exposed to chloride-containing solutions [19]. AgNPs incubated in an aqueous solution of PEG with *M*_n_ = 8000 Da prevented agglomeration in a NaCl concentration between 10 and 100 mM upon increasing the concentration of PEG from 1 to 5 mg/mL. At a PEG concentration as low as 10–100 nM, steric protection is already initiated [21]. Nevertheless, an increase in salt concentration leads to a deterioration in the stabilizing ability of PEG [22].

On the other hand, the topological effects of polymers are gaining attention, and have become another important parameter of polymers in addition to molecular weight [23] and repeating units [24], in recent years. Many researchers have synthesized and successfully isolated polymers with various topological shapes such as monocyclic, multicyclic, H-shape, etc. [25]. Among all topologies, cyclic polymers without end groups exhibit unique physical/chemical properties such as higher glass transition temperature [26], reduced hydrodynamic volume [27], higher density [28], lower viscosity [29], higher resistance of their micelles towards salt [30] and temperature [31], and higher interfacial activity [32] compared with their respective linear counterparts. In particular, we reported that dispersion stability of AuNPs [33] and AgNPs [34] was drastically enhanced by the physisorption of cyclic PEG. With these previous works in mind, the present research investigates the utilization of PEG in the preparation process of AgNPs, where the influences of the linear and cyclic polymer topologies as well as the chain-end groups on the AgNPs’ size and stability against NaCl were investigated.

## 2. Materials and Methods

### 2.1. Materials

Silver nitrate (AgNO_3_) (<99%) and poly(ethylene glycol) (PEG) of *M*_n_ = 2000 Da (HO–PEG_3k_–OH) were purchased from Sigma Aldrich Co., LLC, St. Louis, MO, USA, while dichloromethane (CH_2_Cl_2_), tetrahydrofuran (THF), *n*-heptane, potassium hydroxide (KOH), sodium hydroxide (NaOH), chlorobenzene, maltose monohydrate, sodium chloride (NaCl), ammonia (28–30%), iodomethane (>99.5%), and PEG of *M*_n_ = 4000 Da (HO–PEG_5k_–OH) and 6000 Da (HO–PEG_10k_–OH), were obtained from Kanto Chemicals Co., Inc., Tokyo, Japan.

### 2.2. Instruments

^1^H NMR (400 MHz) and ^13^C NMR (100 MHz) were measured in CDCl_3_ using a JNM-ESC400 instrument (JEOL Ltd., Akishima, Japan) at ambient temperature. SEC was performed at 40 °C in THF (flow rate, 1.0 mL/min), using a Shodex GPC-101 (Showa Denko K.K., Tokyo, Japan) gel permeation chromatography system (Shodex DU-2130 dual pump, Shodex RI-71 reflective index detector, and Shodex ERC-3125SN degasser) equipped with a Shodex KF-G guard column (4.6 mm × 10 mm; pore size, 8 μm) and two Shodex KF-804L columns (8 mm × 300 mm) in series. TEM measurements were performed with a JEM-2010 (JEOL Ltd., Akishima, Japan) operated at 200 kV. The size distribution of AgNPs was determined by the average of 150–200 observed particles. UV–Vis absorption spectra were recorded on a JASCO 4100 spectrophotometer (JASCO Co., Tokyo, Japan) at 25 °C. Relative UV–Vis absorption intensity (*A*/*A*_0_ × 100%) of AgNPs without and with NaCl was compared at *λ*_max_ (409, 405, and 420 nm for HO–PEG_5k_–OH, *c*-PEG_5k_, and MeO–PE*G*_5k_*–*Ome, respectively). MALDI–TOF mass spectrometry was carried out using a Bruker Daltonics Ultraflex-S at the Open Facility, Hokkaido University, Hokkaido, Japan.

### 2.3. Synthesis of c-PEG

HO–PEG_3k_–OH, HO–PEG_5k_–OH, and HO–PEG_10k_–OH were cyclized according to a previous report by Cooke et al. [35]. Typically, HO–PEG_5k_–OH (5.0 g, 1.3 mmol) and TsCl (0.48 g, 2.5 mmol) were dissolved in dry THF (100 mL) and added dropwise to a stirred suspension of KOH (3.3 g) in THF/*n*-heptane (75/25, 100 mL) through a syringe pump, at 40 °C under N_2_. The addition was conducted over 144 h at a rate of 0.7 mL/h. After the addition was concluded, the reaction mixture was stirred for a further 24 h at 40 °C. The resulting suspension was filtered and concentrated in vacuo. Thereafter, the residue was redissolved in CH_2_Cl_2_ and washed with brine once and with distilled water four times, until neutral pH was observed for the aqueous phase. Thereafter, the combined organic phase was concentrated in vacuo, and purification was conducted by fractionation, where the residue was initially dissolved in CH_2_Cl_2_, treated with *n*-heptane until becoming cloudy, heated to 40 °C, and cooled to 25 °C, followed by decantation of the liquid phase from the precipitate. The supernatant was concentrated in vacuo, and the purification step was conducted for a further three times, to give *c*-PEG_5k_ (806 mg, 16%) as a colorless/slightly yellow solid. ^1^H NMR: *δ* (ppm) 3.61 (s, –OC*H*_2_C*H*_2_O–). ^13^C NMR: *δ* (ppm) 70.7 (–O*C*H_2_*C*H_2_O–). *M*_n_(SEC) = 3200 Da, *M*_p_(SEC) = 3200 Da, *M*_w_/*M*_n_ = 1.06.

### 2.4. Synthesis of MeO–PEG–OMe

The dimethylation of HO–PEG_5k_–OH was performed using a previously reported procedure [35]. A suspension of KOH (3.45 g) and iodomethane (2.28 g) in chlorobenzene (20 mL) in a 300 mL three-neck flask was treated dropwise with a solution of HO–PEG_5k_–OH in chlorobenzene (50 mL), over ~20 min. After the addition was completed, the mixture was stirred at 25 °C for 48 h under Ar. The resulting suspension was diluted with CH_2_Cl_2_ and filtered, and the filtrate was concentrated in vacuo. The residue was redissolved in CH_2_Cl_2_ and washed with deionized H_2_O seven times, dried over MgSO_4_ for 24 h, and concentrated in vacuo to obtain MeO–PEG_5k_–OMe (3.38 g, 68%) as a white solid. ^1^H NMR: *δ* (ppm) 3.60 (s, –OC*H*_2_C*H*_2_O–), 3.62 (s, –OCH_2_C*H*_2_OCH_3_), 3.34 (s, –OCH_2_CH_2_OC*H*_3_). ^13^C NMR: *δ* (ppm) 72.0 (–OCH_2_*C*H_2_OCH_3_), 70.7 (–O*C*H_2_*C*H_2_O–), 59.1 (s, –OCH_2_CH_2_O*C*H_3_).

### 2.5. Synthesis of AgNPs through the Tollens’ Process

AgNPs were prepared according to a reported procedure [36]. Thus, to a mixture of AgNO_3_ (17 mg, 0.10 mmol), PEG (10–200 mg, 0.22–4.4 mmol), an aqueous solution of ammonia (28–30%, 34 µL) in deionized H_2_O (90 mL), an aqueous solution of NaOH (10 mL, 0.10 mM) was added, under vigorous stirring. Thereafter, solid maltose monohydrate (360 mg, 1.0 mmol) was added, and the resulting mixture stirred overnight. The prepared AgNPs dispersions were characterized and used without further purification.

## 3. Results

### 3.1. Synthesis of c-PEG

Cyclized PEG with a molecular weight (M_n_) of 3000, 5000, and 10,000 Da (namely, c-PEG_3k_, c-PEG_5k_, and c-PEG_10k_, respectively,) was obtained in a range between 0.7 and 1.5 g, which is a considerably large amount for cyclic polymers to be produced in a single batch reaction. With increasing molecular weight, the cyclization reaction became more difficult to achieve, which led to a decrease in the yield (Table S1) [35]. In this work, HO–PEG–OH was cyclized via etherification through the modified tosylation reaction, in line with previous reports [37]. Thus, formed cyclic PEG was homogeneous in the chemical structure. Recently, Hirose et al. reported the preparation of cyclic PEG (M_n_ = 400–1000 Da), where the two terminal groups of the polymer are connected by a C_12_ chain [38]. Additionally, the cyclization of PEG can also be achieved by performing the Glaser coupling [39] or via click chemistry [40]. However, the mentioned reactions are limited PEG with low M_n_ or conducted in batch reactions under high dilutions, which often yield a low amount of product (less than 100 mg product per batch), thus rendering them unattractive for our research, since larger amounts of polymer are required for performing sufficient tests. Each single peak of size-exclusion-chromatography (SEC) trace exhibits the high purity of HO–PEG–OH and c-PEG (Figure S1). The lower molecule weight-shift of c-PEG in the SEC traces, reflects the reduced hydrodynamic volume of polymers upon cyclization. For instance, M_p_ = 3100 of HO–PEG_3k_–OH was decreased to 2000 upon the polymer topology conversion. Furthermore, matrix-assisted laser desorption ionization time-of-flight (MALDI-TOF) mass spectra of HO–PEG_3k_–OH showed a peak at m/z = 2022.74 (DP_n_ = 45, Na^+^ adduct), whereas the corresponding peak from c-PEG_3k_ was at m/z = 2005.02 (Figure S2); the value of the highest peak in the isotope distribution shifted by a mass unit of 18, due to the net elimination of a water molecule upon cyclization. This was also observed for c-PEG_5k_ (Figure S3), but it was not possible to conduct the same MALDI-TOF mass measurements for c-PEG_10k_, due to its large M_n_. Moreover, ^13^C nuclear magnetic resonance (NMR) of c-PEG showed the complete disappearance of the carbon atoms adjacent to the terminal hydroxy groups (~61.6 ppm) in HO–PEG–OH, which confirmed the effective elimination of the terminal groups (Figures S4–S6).

### 3.2. Synthesis of AgNPs

AgNPs were prepared by the Tollens’ method, similar to the report by Kvítek et al., with a modification which consisted of using PEG instead of poly(acrylic acid) as a polymeric stabilizer [36]. In this set of experiments, the molar ratio of PEG to AgNO_3_ (ω) was varied from 2.2 to 44, while other reaction parameters were kept constant (Table 1). Hydroxy-terminated linear PEG with M_n_ of 3000, 5000 and 10,000 Da, HO–PEG_3k_–OH, HO–PEG_5k_–OH, and HO–PEG_10k_–OH, respectively, as well as their corresponding cyclic counterparts, c-PEG_3k_, c-PEG_5k_, and c-PEG_10k_, respectively, were used as a polymeric stabilizer.

**Figure 1:**
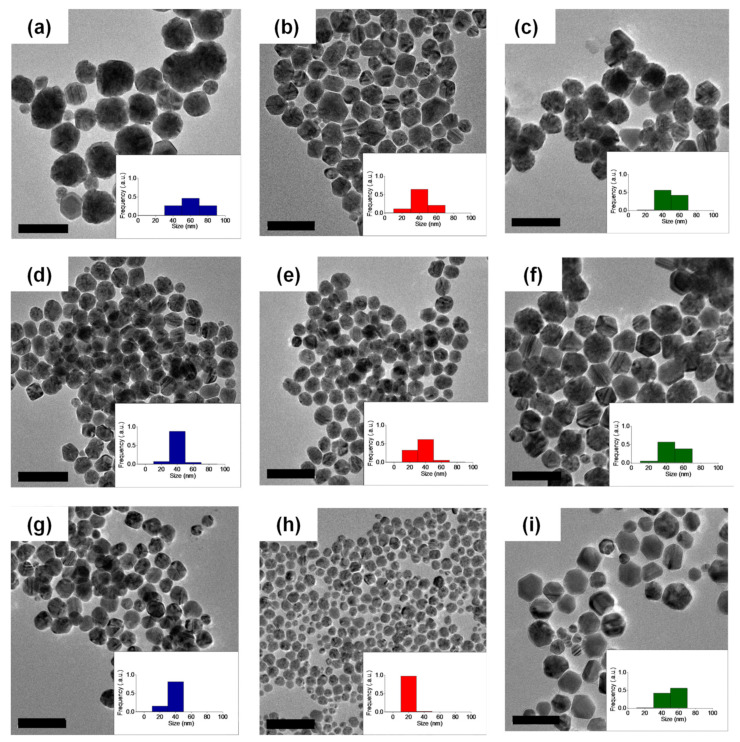
TEM micrographs of AgNPs prepared in the presence of (**a**) HO–PEG_5k_–OH (*ω* = 2.2), (**b**) *c*-PEG_5k_ (*ω* = 2.2), (**c**) MeO–PEG_5k_–OMe (*ω* = 2.2), (**d**) HO–PEG_5k_–OH (*ω* = 11), (**e**) *c*-PEG_5k_ (*ω* = 11), (**f**) MeO–PEG_5k_–OMe (*ω* = 11), (**g**) HO–PEG_5k_–OH (*ω* = 44), (**h**) *c*-PEG_5k_ (*ω* = 44), and (**i**) MeO–PEG_5k_–OMe (*ω* = 44) (Scale bar: 100 nm).

**Figure 2:**
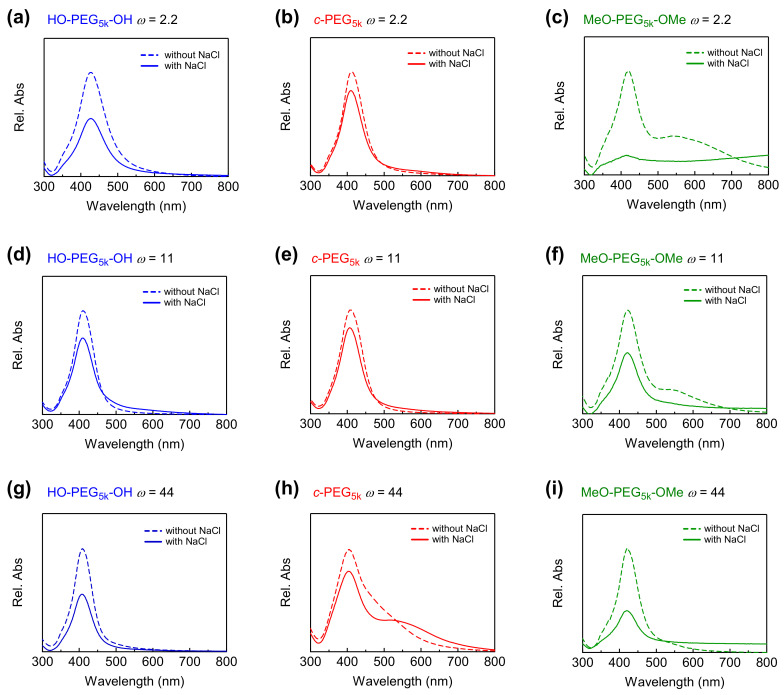
Relative UV–Vis absorption spectra of AgNPs without NaCl (dashed line) and with 37.5 mM of NaCl (solid line) in the presence of (**a**) HO–PEG_5k_–OH, (**b**) *c*-PEG_5k_, (**c**) MeO–PEG_5k_–OMe at *ω* = 2.2, (**d**) HO–PEG_5k_–OH, (**e**) *c*-PEG_5k_, (**f**) MeO–PEG_5k_–OMe at *ω* = 11, (**g**) HO–PEG_5k_–OH, (**h**) *c*-PEG_5k_, and (**i**) MeO–PEG_5k_–OMe at *ω* = 44.

In addition, linear PEG (M_n_ = 5000) with methoxy termini (MeO–PEG_5k_–OMe) was also tested to investigate the effects of the end groups. AgNPs that were prepared in this work featured a similar particle size spectrum to the observation made by Kvítek et al. at comparable polymer concentrations [36]. Here, PEG was primarily used as a stabilizing agent, while the reducing environment was provided through the use of maltose monohydrate. The pH of the reaction was adjusted to ~11 by using NaOH, which was responsible for the completion of the reaction within minutes. The formed particles were characterized by transmission electron microscopy (TEM) and UV–Vis spectroscopy (Figure 1, Figure 2 and Figures S7–S13).

The particle size obtained from the experiments using c-PEG_5k_ and c-PEG_10k_ was significantly smaller, compared with the cases of corresponding HO–PEG_5k_–OH and HO–PEG_10k_–OH, respectively, (Table 1, Figure 1 and Figures S10–S12). For example, at ω = 44, 21 ± 4 nm for c-PEG_5k_ was substantially smaller than 40 ± 7 nm for HO–PEG_5k_–OH. Nonetheless, the average particle size remained similar when HO–PEG_3k_–OH and c-PEG_3k_ were used at the respective ω values (Table 1, Figures S7–S9). For example, at ω = 44, 30 ± 7 nm for c-PEG_3k_ was comparable to 33 ± 4 nm for HO–PEG_3k_–OH. On the other hand, an increase in the ω value using a same type of polymer generally led to a decrease in the average particle size. Taking c-PEG_5k_ as an example, the size decreased from 42 ± 9 nm at ω = 2.2 to 31 ± 4 nm at ω = 11, and eventually to 21 ± 4 nm at ω = 44. These results likely arose from an increase in the potential interaction sites between the silver ions (in the form of [Ag(NH_3_)]_2_^+^) and the PEG chain, suppressing the growth of the nanoparticles. The oxyethylene units of PEG complexed with the Ag^+^ ions, which were then reduced to form particles [41]. Initially, primary nanoparticles were formed, which underwent coalescence with their neighboring species to form the more stable secondary nanoparticles. In this regard, Shin et al. stated that when the concentration of the polymer is increased, the number of primary AgNPs at close range decreases, thus reducing the possibility of coalescence and eventually yielding small particle sizes [42].

The increase in the ω value meant that it is accompanied by the increase of the number of the hydroxy end groups for HO–PEG–OH, while no hydroxy end groups existed in c-PEG. In order to eliminate the effects of the hydroxy end groups, to determine the strict topology effects, PEG with methoxy end groups (MeO–PEG_5k_–OMe) was synthesized and subjected to the same AgNPs formation experiment. Interestingly, the size of AgNPs formed in the presence of MeO–PEG_5k_–OMe was basically unchanged (44–48 nm), despite the ω value (Table 1, Figure 1c,f,i). This result suggested that the increase in the concentration of the hydroxy end groups leads to the decrease in the particle size, and this effect plays a significant role in the nanoparticle size, using HO–PEG–OH. Therefore, the strict topology effects were actually more prominent by comparing the AgNPs sizes of c-PEG_5k_ and MeO–PEG_5k_–OMe; 21 ± 4 nm for c-PEG_5k_ and 48 ± 9 nm for MeO–PEG_5k_–OMe at ω = 44 as a representative example. In our opinion, the smaller hydrodynamic volume and the corresponding denser structure of c-PEG, similar to the case of the micelles from amphiphilic block copolymers [43,44], provided more effective shielding of the metal surface from the coalescence of the primary nanoparticles, which led to the reduction of the average particle size.

The size of the AgNPs was also characterized by UV–Vis spectroscopy (Figure 2 and Figure S13, dotted lines). For example, at ω = 44, the absorption maximum (λ_max_) was found at 409, 405, and 420 nm for HO–PEG_5k_–OH, c-PEG_5k_, and MeO–PEG_5k_–OMe, respectively. The correlation of the mean particle diameter measured by TEM with λ_max_ in the UV−Vis spectrum was reported previously [45], and the present results reasonably matched the report (Figure S14). In the meantime, broadened UV–Vis absorption in the longer wavelengths was observed for some samples such as c-PEG_5k_ at ω = 44 (Figure 2h), MeO–PEG_5k_–OMe at ω = 2.2 and 11 (Figure 2c,f). According to Tadano et al., the polymers can sometimes cause the interactions (i.e., hydrogen bonding and van der Waals interaction) between the polymer chains, which eventually trigger the agglomeration of the nanoparticles [46]. The extent of the agglomeration, however, is also dependent on the particle size and surface properties [47]. At this point, we have not determined the cause of the broadening trend by ω, the molecular weight, or the topology of the polymer. Nevertheless, this effect also manifested itself in the corresponding UV–Vis spectra, where absorption at higher wavelengths appeared. Dynamic light scattering was also attempted for size characterization. However, no valuable data were obtained, likely due to the low concentration of the particles, which gave relatively weak and disturbed signals, where the appropriate concentration of samples for DLS is normally in the range of 1–10 mg/mL.

### 3.3. Stability of AgNPs

**Figure 3:**
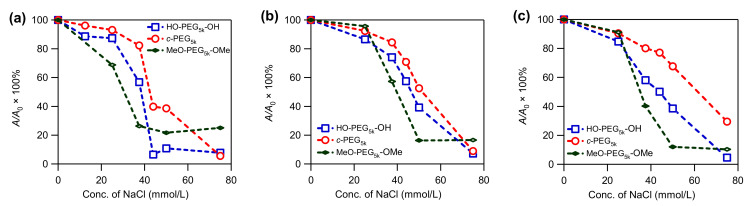
Plots of relative UV–Vis absorption intensity (*A/A*_0_ × 100%) at *λ*_max_ versus the NaCl concentration for AgNPs prepared in the presence of HO–PEG_5k_–OH (blue), *c*-PEG_5k_ (red), and MeO–PEG_5k_–OMe (green) at (**a**) *ω* = 2.2, (**b**) *ω* = 11, and (**c**) *ω* = 44.

The prepared AgNPs were subjected to stability tests by exposing them to a solution containing various concentrations of NaCl. The effect of the salt on the intensity of the absorption at λ_max_ was monitored by UV–Vis spectroscopy. The AgNPs prepared in the absence of PEG were susceptible to NaCl, and completely lost the absorption at the salt concentration of 37.5 mM, likely due to aggregation and dissolution (Figure S13, solid line). In comparison, AgNPs prepared in the presence of various PEG were more stable. Among them, AgNPs stabilized by c-PEG exhibited a higher resistance, compared with those stabilized by corresponding HO–PEG–OH. This phenomenon was illustrated by the c-PEG-stabilized AgNPs’ ability to preserve the relative UV–Vis absorption intensity (A/A_0_ × 100%) at λ_max_ more efficiently than their linear counterparts, especially at the NaCl concentration range from 37.5 to 50 mM (Figure 2 and Figure 3 and Figures S15–S18). For example, at ω = 44 with a NaCl concentration of 37.5 mM, the relative absorption for AgNPs with HO–PEG_5k_–OH (blue) was 58%, while for c-PEG_5k_ (red) it was 80% (Figure 3c). Moreover, for MeO–PEG_5k_–OMe (green), it was only 40% under the same conditions, suggesting the hydroxy end groups also played an important role in the stabilization against NaCl, due to the PEG and AgNPs surface interactions. It is likely that the hydroxy end groups bound to the surface of the AgNPs and protected them from corrosion with chloride anions. Thus, the pure topology effects which were not caused by the hydroxy end groups, on the stabilization of the AgNPs, can be considered as the difference between c-PEG_5k_ (80%) and MeO–PEG_5k_–OMe (40%), despite the fact that AgNPs with c-PEG_5k_ were much smaller (21 ± 4 nm) than those with MeO–PEG_5k_–OMe (48 ± 9 nm). In the meantime, the difference between HO–PEG_3k_–OH and c-PEG_3k_ was not as significant, except for ω = 44 (Figure S17), suggesting that the molecular weight and the ω value were also essential factors.

## 4. Conclusions

In conclusion, it has been shown that c-PEG tends to yield smaller and more narrowly dispersed AgNPs when employed during Tollens’ synthesis in comparison with HO–PEG–OH and MeO–PEG–OMe, where smaller and narrowly dispersed metal nanoparticles are desired in various applications. Furthermore, the AgNPs with c-PEG exhibited superior stabilizing properties against NaCl, despite the smaller size. Specifically, the pure topological conversion resulted in the drastically improved persistence of the UV–Vis absorption intensity (c-PEG_5k_, 80%; MeO–PEG_5k_–OMe, 40%). These results clearly indicate that cyclized polymers endow AgNPs with superior stability, compared with their linear counterparts. The enhanced colloidal stability through the use of c-PEG would enable applications within biological systems, where the high salt resistance can be exploited. This advantage is coupled with the biocompatibility of PEG itself, where cyclization requires no chemical functionalization on the repeating units of the polymer chain.

## Figures and Tables

**Table 1:** Summary of the TEM-Measured Size of AgNPs Prepared in the Presence of HO–PEG–OH, *c*-PEG, and MeO–PEG–OMe with Various Molecular Weight and *ω*.

Polymer	*ω* ^1^	TEM-Measured Size of AgNPs (nm)		
		HO–PEG–OH	*c*-PEG	MeO–PEG–OMe
PEG_3k_	2.2	55 ± 8	52 ± 8	-
	11	43 ± 8	45 ± 8	-
	44	33 ± 4	30 ± 7	-
PEG_5k_	2.2	58 ± 13	42 ± 9	48 ± 6
	11	36 ± 5	31 ± 4	44 ± 10
	44	40 ± 7	21 ± 4	48 ± 9
PEG_10k_	2.2	50 ± 8	30 ± 3	-
	11	48 ± 10	35 ± 4	-
	44	37 ± 7	26 ± 4	-

^1^*ω* is given by the molar ratio of PEG repeating units to AgNO_3_ used in the preparation of AgNPs.

## Data Availability

The data presented in this study are available on request from the corresponding author.

## Supplementary Materials

The following supporting information can be downloaded at: https://www.mdpi.com/article/10.3390/polym14214535/s1, Table S1: Molecular weight of PEG samples, Figure S1: SEC traces of PEG samples, Figures S2 and S3: MALDI-TOF spectra of PEG samples, Figures S4–S6: NMR spectra of PEG samples, Figures S7–S12: TEM micrographs of AgNPs, Figure S13: UV–Vis absorption spectra of AgNPs, Figure S14: Correlation between the particle size of AgNPs by TEM analysis and UV-Vis spectroscopy, Figures S15 and S16: UV–Vis absorption spectra of AgNPs. Figures S17 and S18: Plots of relative UV–Vis absorption intensity versus the NaCl concentration.
