# Supplementary material for: Size Control and Enhanced Stability of Silver Nanoparticles by Cyclic Poly(ethylene glycol)
Source: Polymers (Basel). 2022 Oct 26;14(21):4535. doi: 10.3390/polym14214535 (PMC9657728; doi:10.3390/polym14214535)
Supplement: Supplementary file 1 [file polymers-14-04535-s001.zip › polymers-1996901-supplementary.pdf]

# Supplementary Materials

# Size Control and Enhanced Stability of Silver Nanoparticles by Cyclic Poly(Ethylene Glycol)

**Yubo Wang [1,†], Jose Enrico Quijano Quinsaat [2,†], Feng Li [2], Takuya Isono [2], Kenji Tajima [2], Toshifumi Satoh [2], Shin-ichiro Sato [2] and Takuya Yamamoto [2,*]**

[1] Graduate School of Chemical Sciences and Engineering, Hokkaido University, Sapporo 060-8628, Hokkaido, Japan
[2] Division of Applied Chemistry, Faculty of Engineering, Hokkaido University, Sapporo 060-8628, Hokkaido, Japan
* Correspondence: yamamoto.t@eng.hokudai.ac.jp
† These authors contributed equally to this work.

**Table S1. Peak Top Molecular Weights ($M_p$) and Polydispersity Index ($M_w/M_n$) of HO–PEG–OH (before cyclization) and *c*-PEG (after cyclization) determined by SEC and Their Percent Yields.**

| | **HO–PEG–OH** | | ***c*-PEG** | | yield (%) |
|---|---|---|---|---|---|
| | $M_p$ (Da) | $M_w/M_n$ | $M_p$ (Da) | $M_w/M_n$ | |
| **$PEG_{3k}$** | 3,100 | 1.06 | 2,000 | 1.10 | 29 |
| **$PEG_{5k}$** | 4,600 | 1.04 | 3,200 | 1.06 | 16 |
| **$PEG_{10k}$** | 14,700 | 1.03 | 10,500 | 1.03 | 14 |

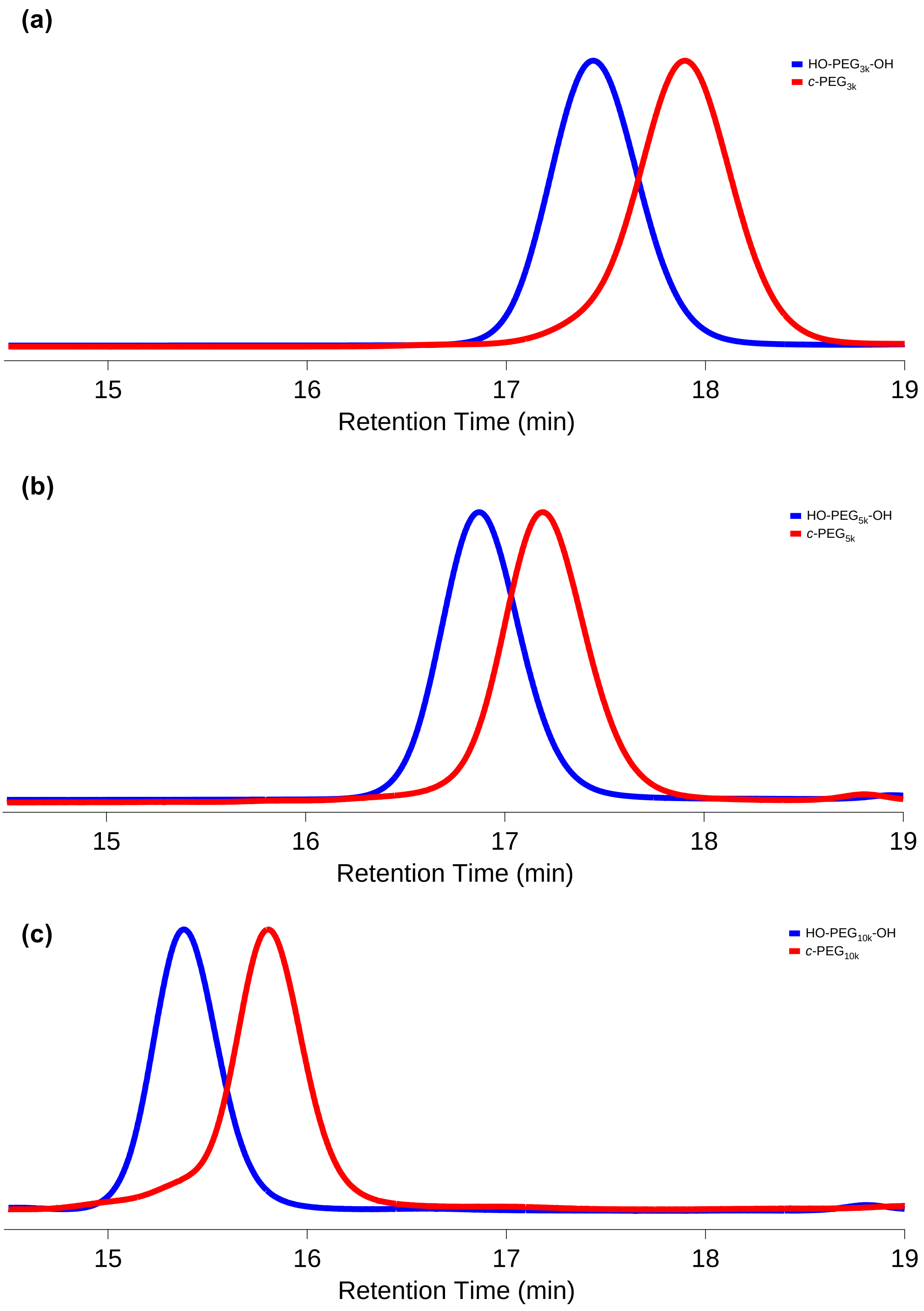


**Figure S1.** SEC traces of (a) HO–PEG$_{3k}$–OH (blue), *c*-PEG$_{3k}$ (red), (b) HO–PEG$_{5k}$–OH, *c*-PEG$_{5k}$, and (c) HO–PEG$_{10k}$–OH, *c*-PEG$_{10k}$.

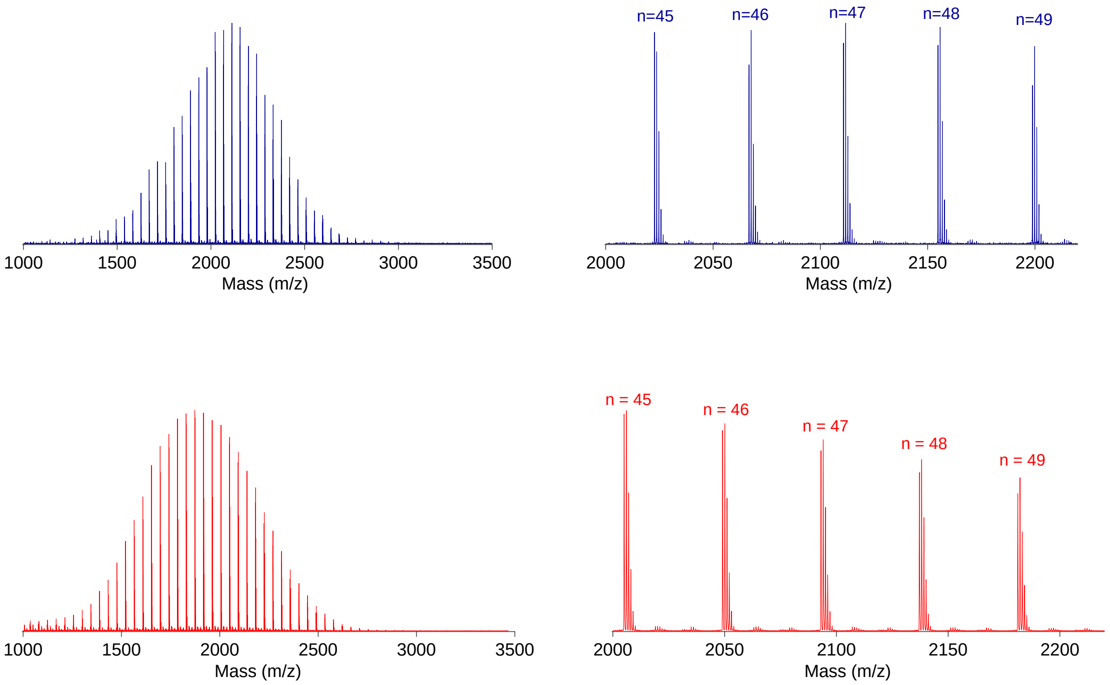


**Figure S2.** MALDI-TOF spectra of HO–$PEG_{3k}$–OH (blue) and *c*-$PEG_{3k}$ (red). $Na^+$ adducts appeared as the main series of the peaks.

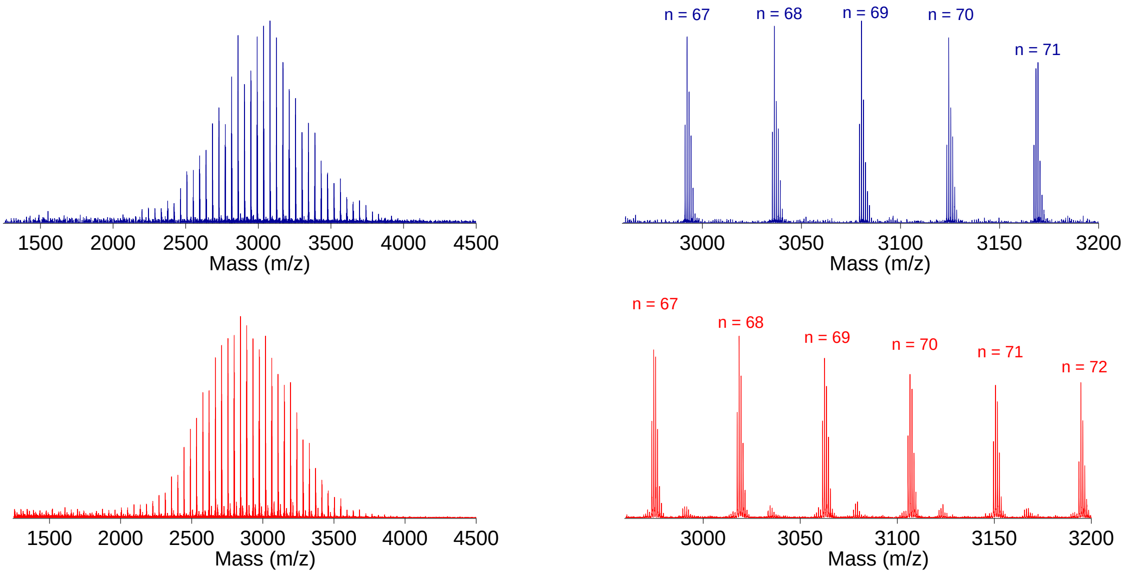


**Figure S3.** MALDI-TOF spectra of HO–$PEG_{5k}$–OH (blue) and *c*-$PEG_{5k}$ (red). $Na^+$ adducts appeared as the main series of the peaks. $K^+$ adducts were also observed in *c*-$PEG_{5k}$.

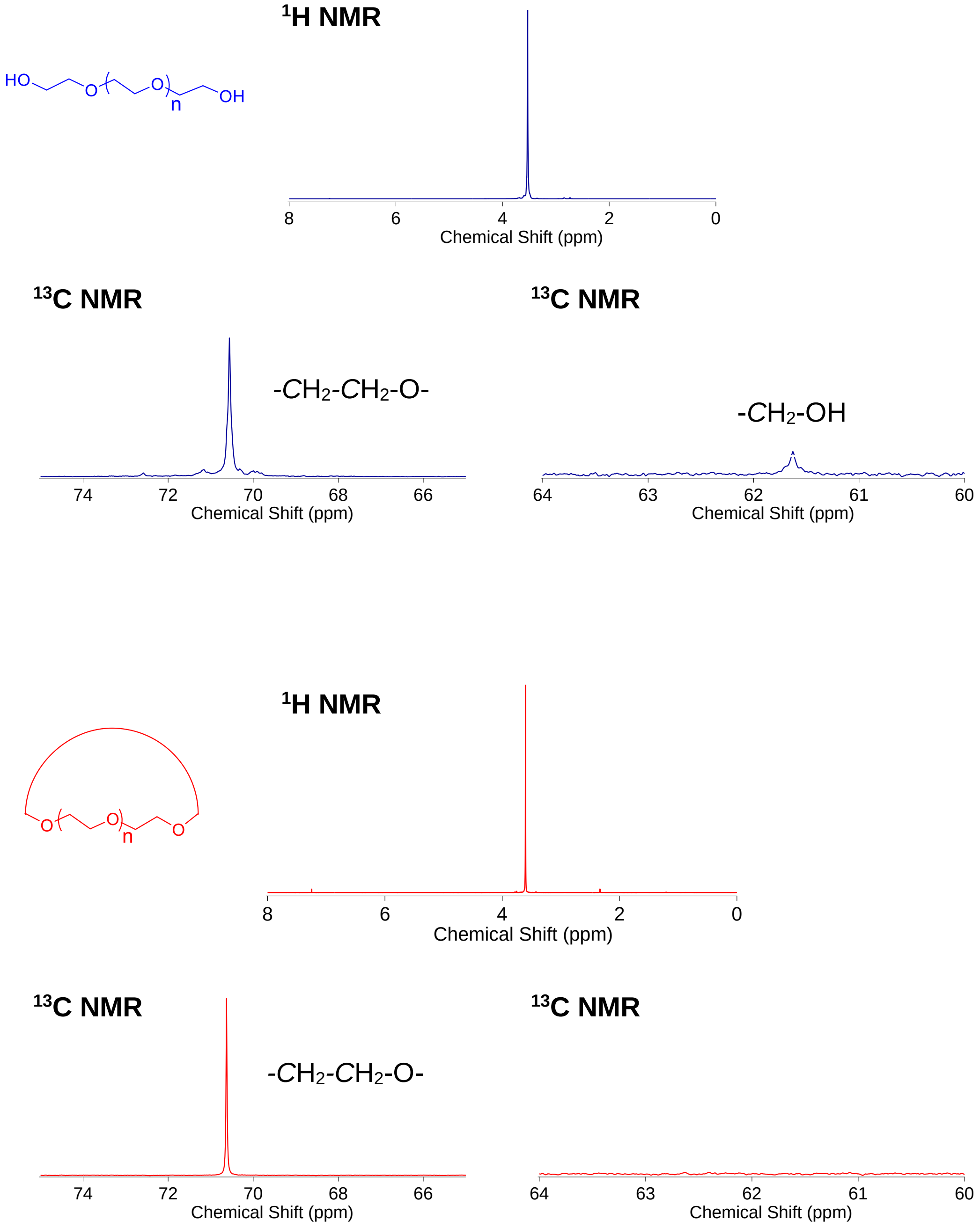


**Figure S4.** $^{1}$H and $^{13}$C NMR spectra of HO–PEG$_{3k}$–OH (blue) and *c*-PEG$_{3k}$ (red).

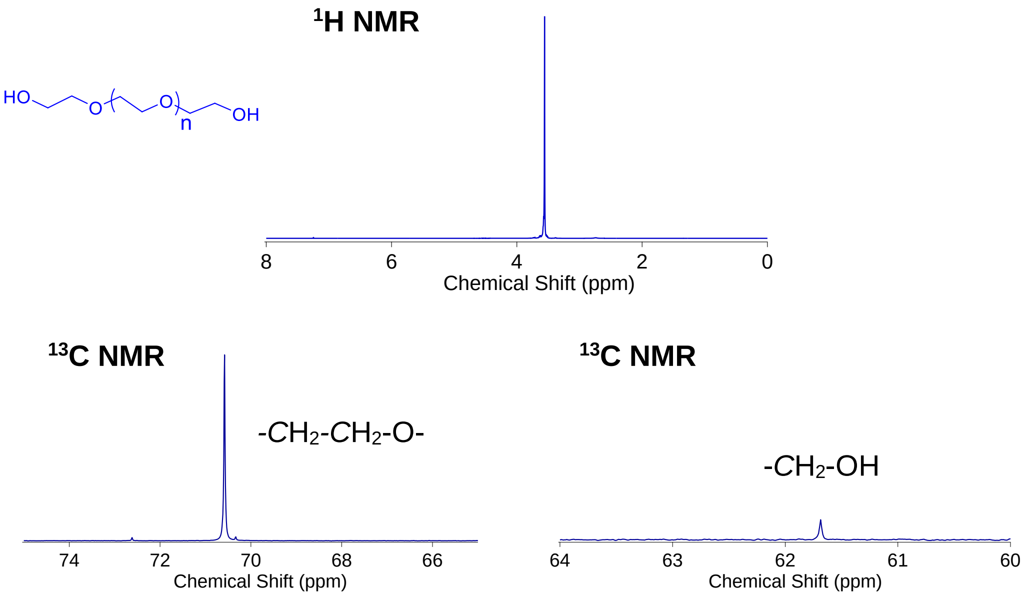
HO
O
O
n
OH
1H NMR
8
6
4
2
0
Chemical Shift (ppm)
13C NMR
-CH2-CH2-O-
74
72
70
68
66
Chemical Shift (ppm)
13C NMR
-CH2-OH
64
63
62
61
60
Chemical Shift (ppm)

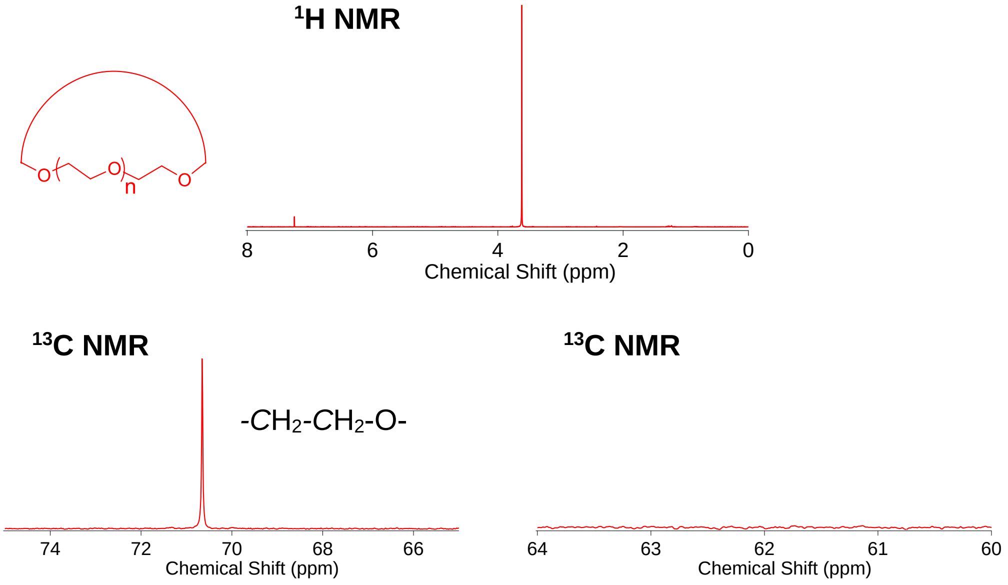
1H NMR
O
O
n
O
8
6
4
2
0
Chemical Shift (ppm)
13C NMR
-CH2-CH2-O-
74
72
70
68
66
Chemical Shift (ppm)
13C NMR
64
63
62
61
60
Chemical Shift (ppm)

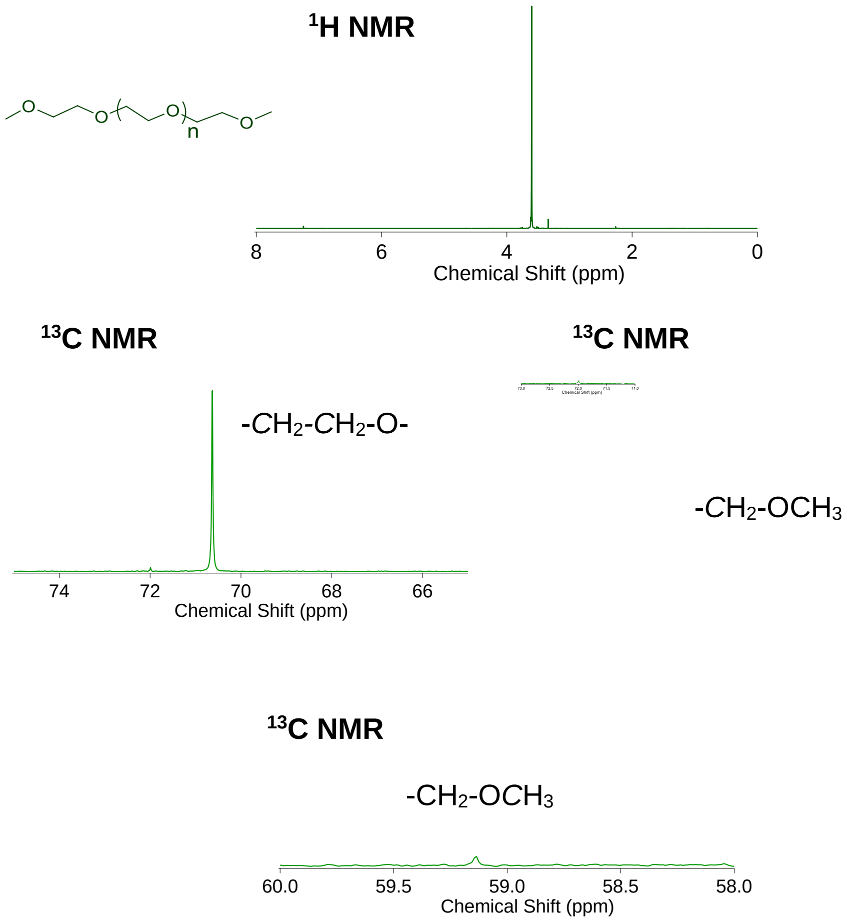


**Figure S5.** $^1$H and $^{13}$C NMR spectra of HO–PEG$_{5k}$–OH (blue), *c*-PEG$_{5k}$ (red), and MeO–PEG$_{5k}$–OMe (green).

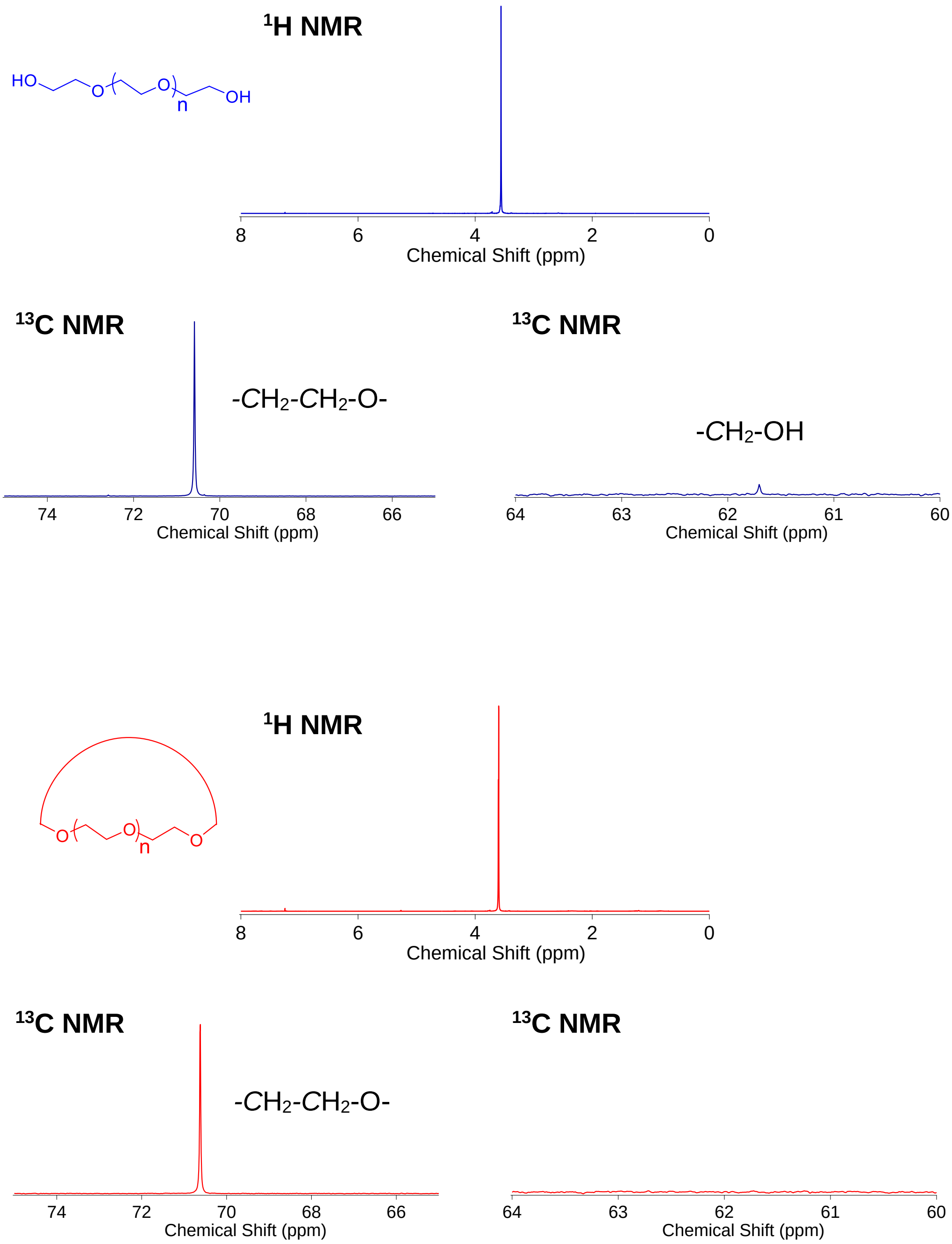


**Figure S6.** $^1$H and $^{13}$C NMR spectra of HO–PEG$_{10k}$–OH (blue) and *c*-PEG$_{10k}$ (red).

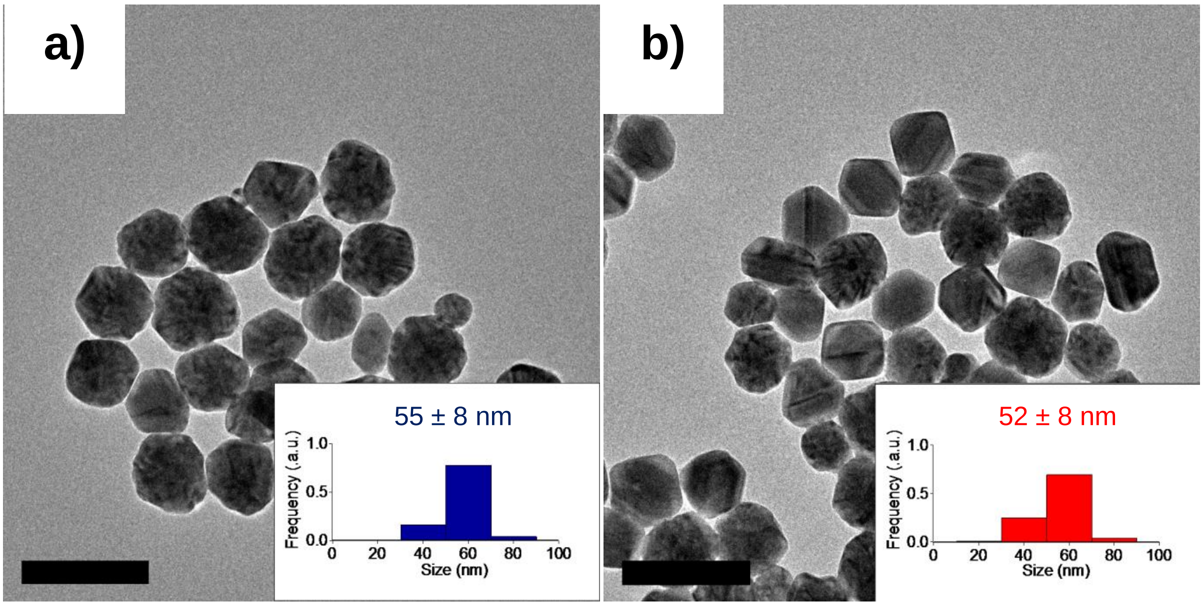


**Figure S7.** TEM micrographs of AgNPs prepared in the presence of (a) HO–PEG$_{3k}$–OH and (b) *c*-PEG$_{3k}$ at $\omega$ = 2.2 (Scale bar: 100 nm).

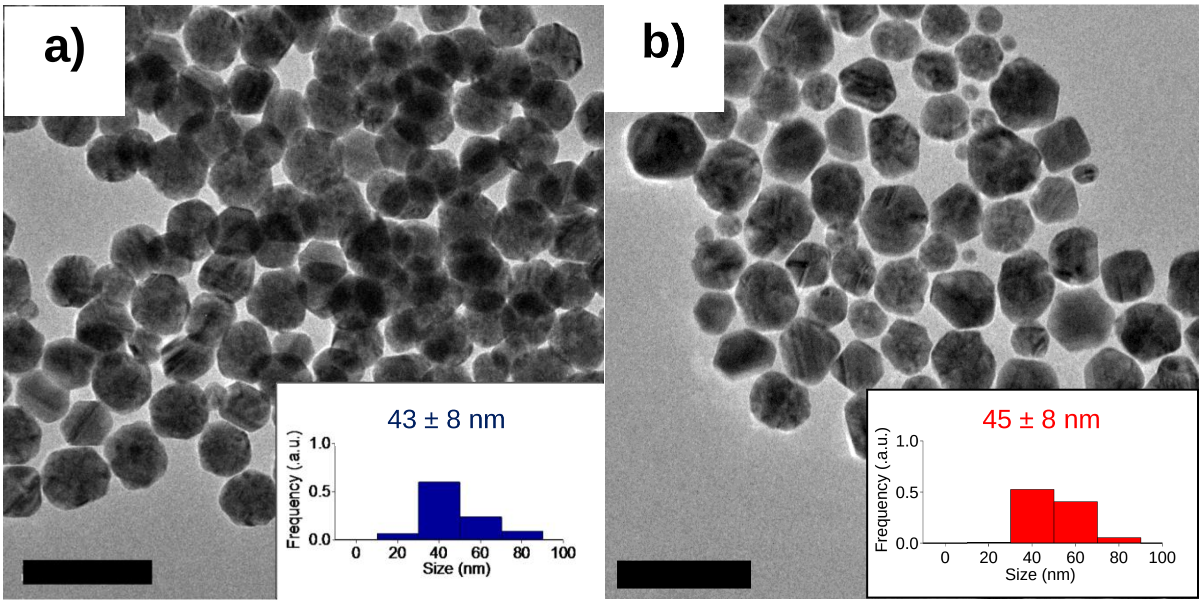


**Figure S8.** TEM micrographs of AgNPs prepared in the presence of (a) HO–PEG$_{3k}$–OH and (b) *c*-PEG$_{3k}$ at $\omega$ = 11 (Scale bar: 100 nm).

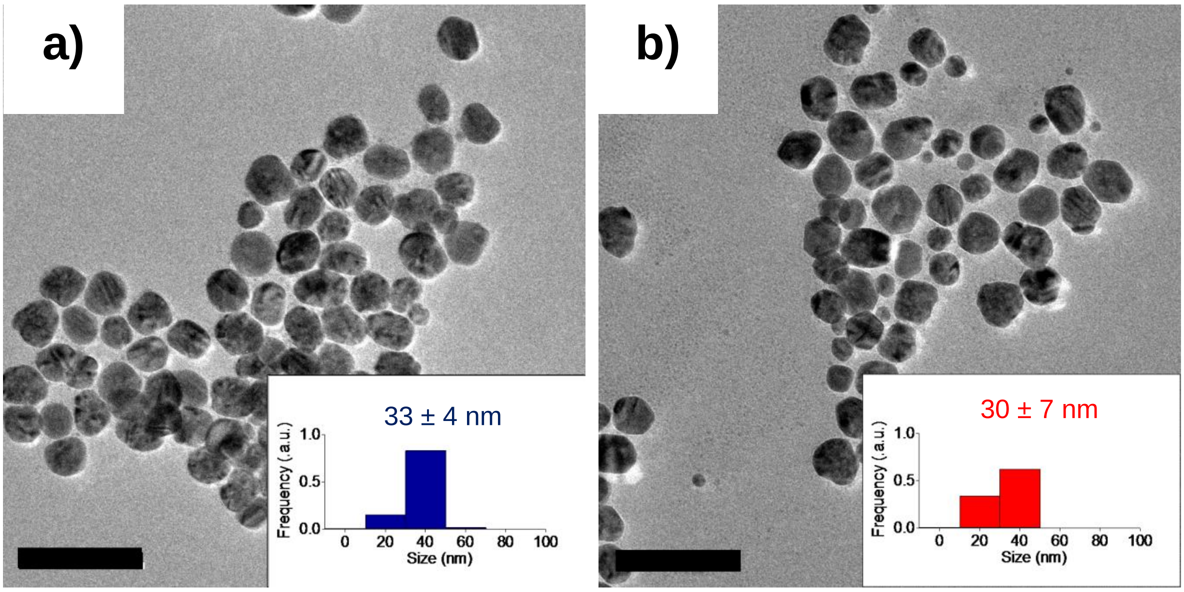


**Figure S9.** TEM micrographs of AgNPs prepared in the presence of (a) HO–PEG$_{3k}$–OH and (b) *c*-PEG$_{3k}$ at $\omega$ = 44 (Scale bar: 100 nm).

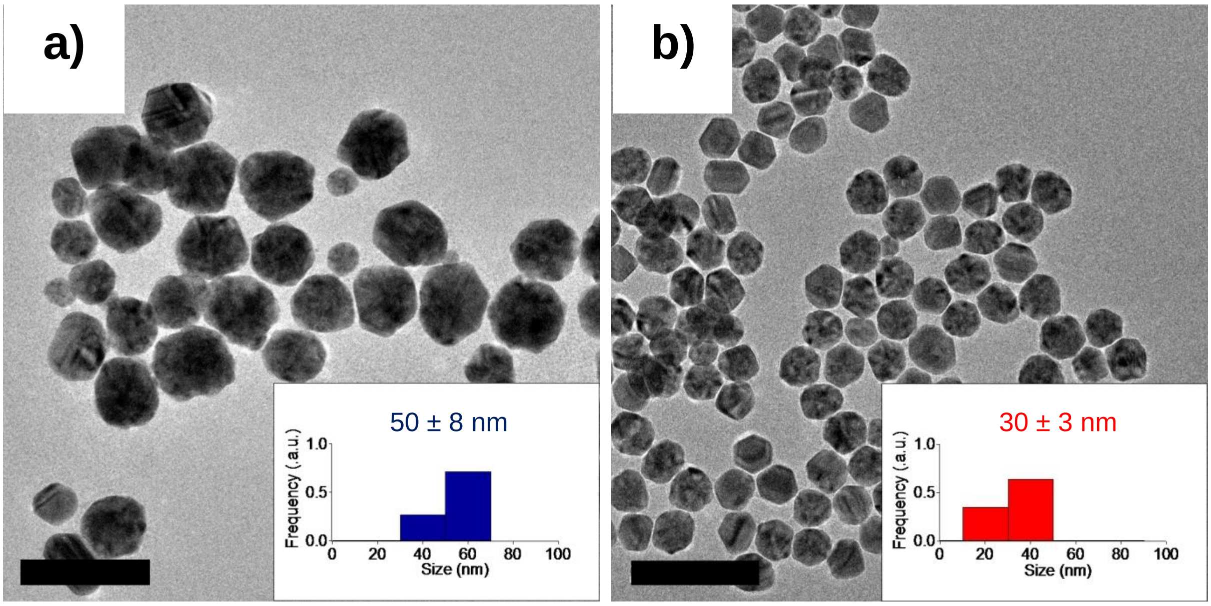


**Figure S10.** TEM micrographs of AgNPs prepared in the presence of (a) HO–PEG$_{10k}$–OH and (b) *c*-PEG$_{10k}$ at $\omega$ = 2.2 (Scale bar: 100 nm).

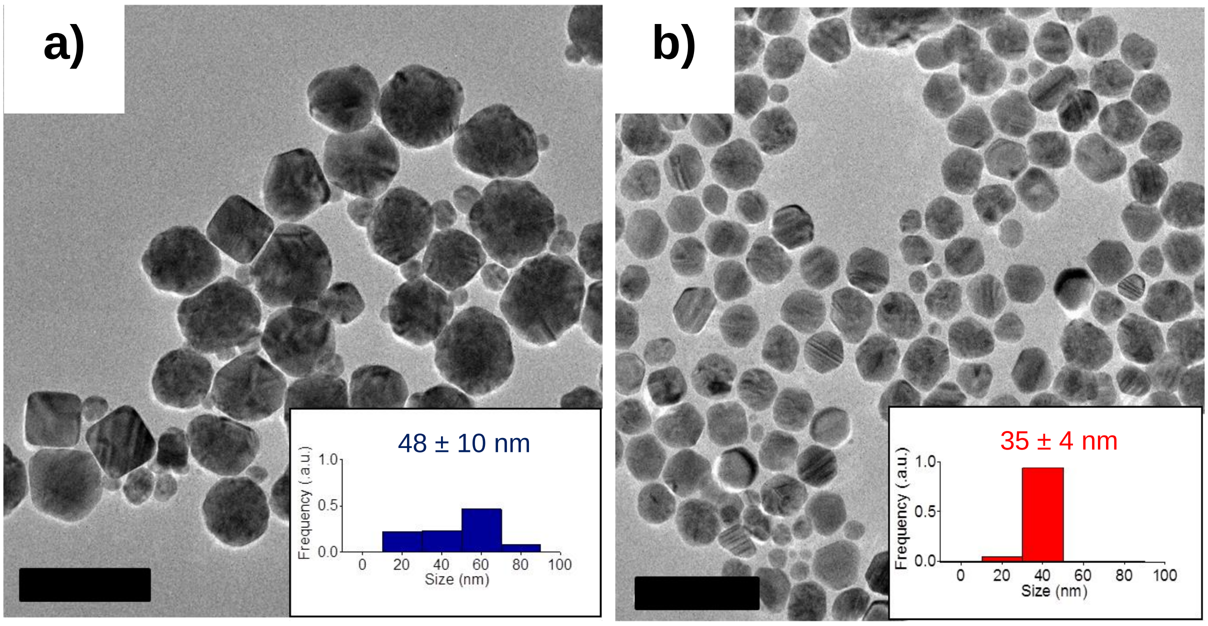


**Figure S11.** TEM micrographs of AgNPs prepared in the presence of (a) HO–$PEG_{10k}$–OH and (b) *c*-$PEG_{10k}$ at $\omega = 11$ (Scale bar: 100 nm).

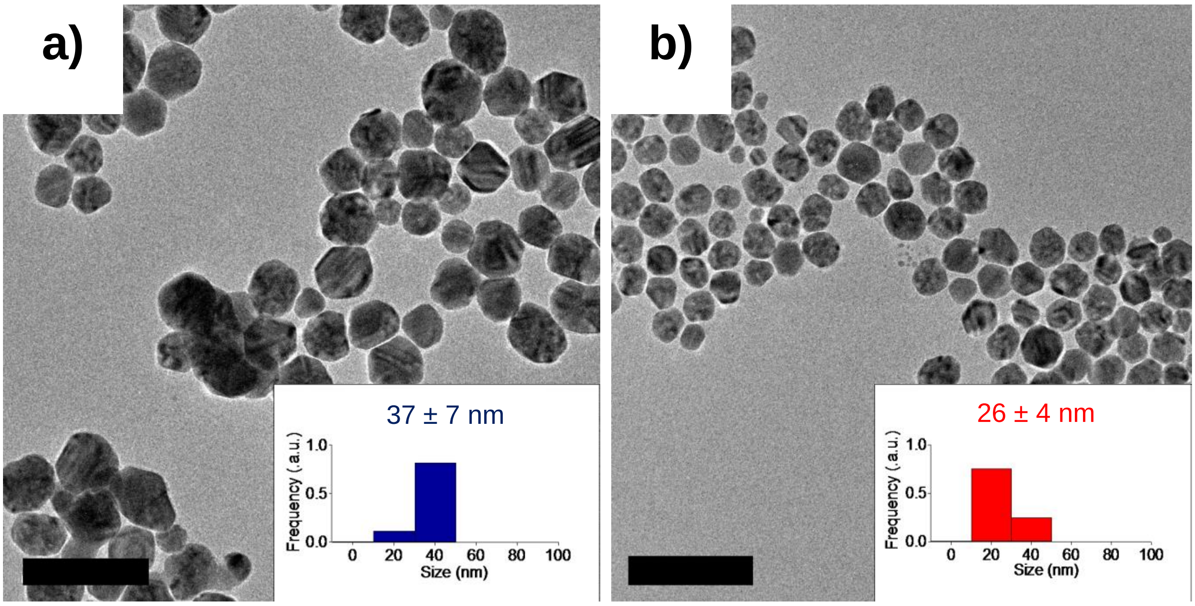


**Figure S12.** TEM micrographs of AgNPs prepared in the presence of (a) HO–$PEG_{10k}$–OH and (b) *c*-$PEG_{10k}$ at $\omega = 44$ (Scale bar: 100 nm).

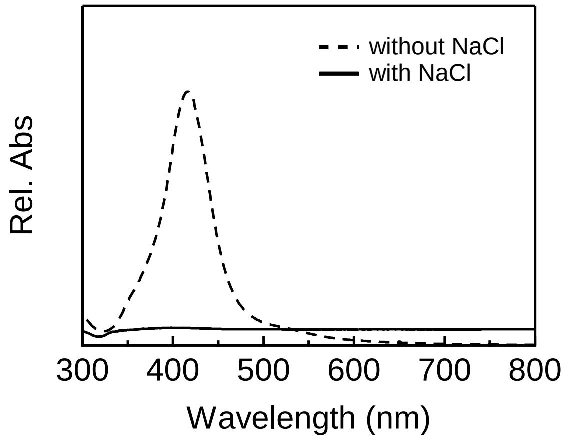


**Figure S13.** Relative UV–Vis absorption spectra of AgNPs without NaCl (dashed line) or with 37.5 mM of NaCl (solid line) in the absence of PEG.

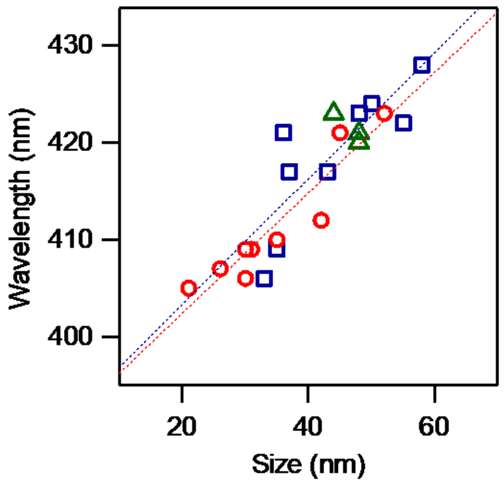


**Figure S14.** Correlation between the particle size of AgNPs extracted from TEM analysis and their $\lambda_{max}$ measured by UV–Vis spectroscopy for HO–PEG–OH (blue square), *c*-PEG (red circle), and MeO–PEG–OMe (green triangle). The linear regression lines calculated for ones prepared with HO–PEG–OH (blue) and *c*-PEG (red) are included in the plot.

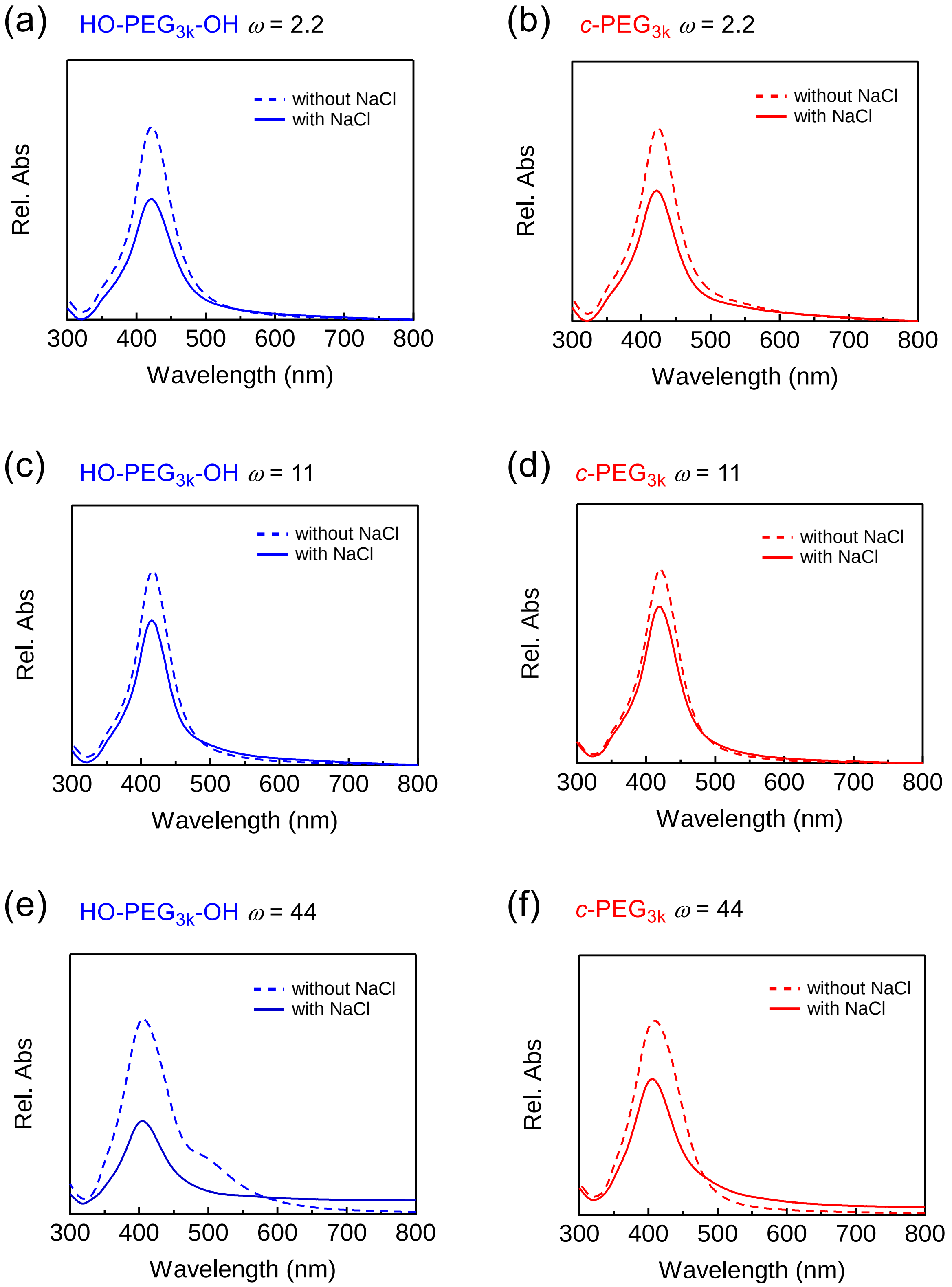


**Figure S15.** Relative UV–Vis absorption spectra of AgNPs without NaCl (dashed line) and with 37.5 mM of NaCl (solid line) in the presence of (a) HO–PEG$_{3k}$–OH and (b) *c*-PEG$_{3k}$ at $\omega$ = 2.2, (c) HO–PEG$_{3k}$–OH and (d) *c*-PEG$_{3k}$ at $\omega$ = 11, (e) HO–PEG$_{3k}$–OH and (f) *c*-PEG$_{3k}$ at $\omega$ = 44.

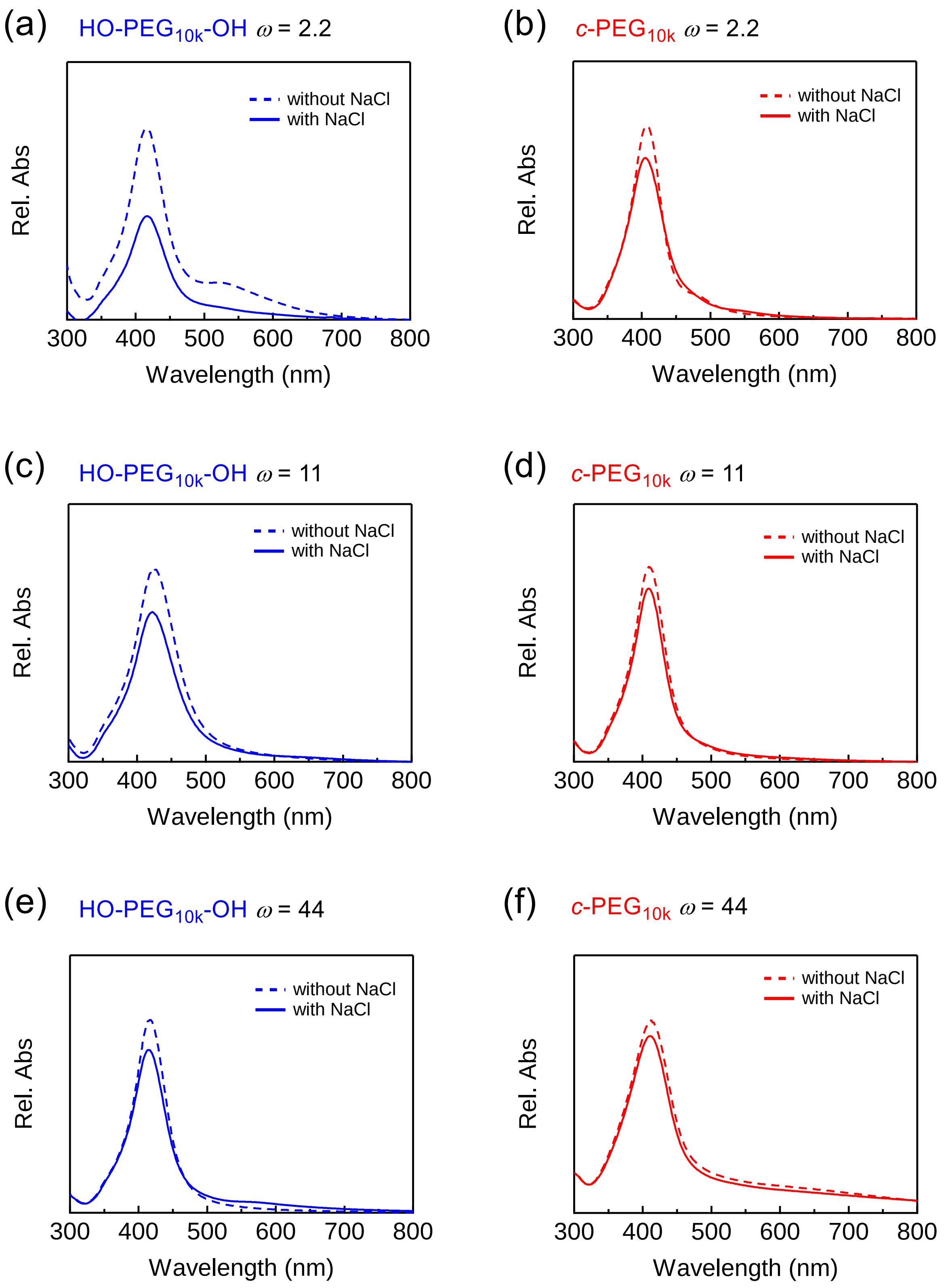


**Figure S16.** Relative UV–Vis absorption spectra of AgNPs without NaCl (dashed line) and with 37.5 mM of NaCl (solid line) in the presence of (a) HO–PEG$_{10k}$–OH and (b) *c*-PEG$_{10k}$ at $\omega$ = 2.2, (c) HO–PEG$_{10k}$–OH and (d) *c*-PEG$_{10k}$ at $\omega$ = 11, (e) HO–PEG$_{10k}$–OH and (f) *c*-PEG$_{10k}$ at $\omega$ = 44.

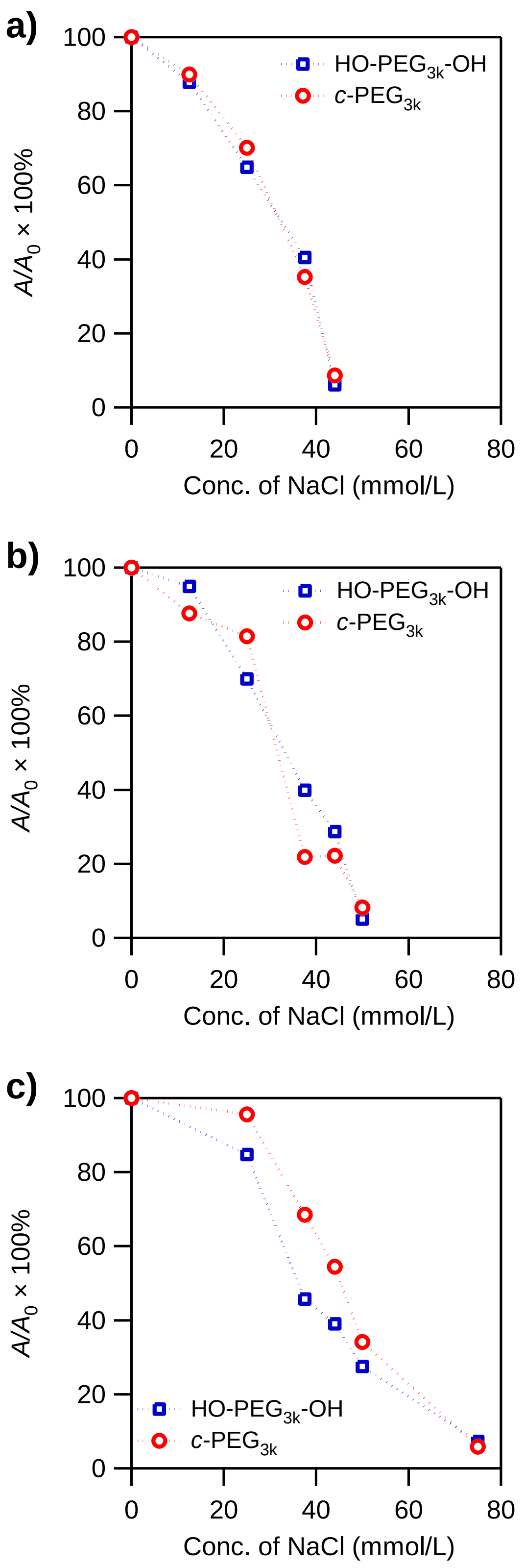


**Figure S17.** Plots of relative UV–Vis absorption intensity ($A/A_0 \times 100\%$) at $\lambda_{max}$ versus the NaCl concentration for the AgNPs prepared in the presence of HO–PEG$_{3k}$–OH (blue) and *c*-PEG$_{3k}$ (red) at (a) $\omega = 2.2$, (b) $\omega = 11$, and (c) $\omega = 44$.

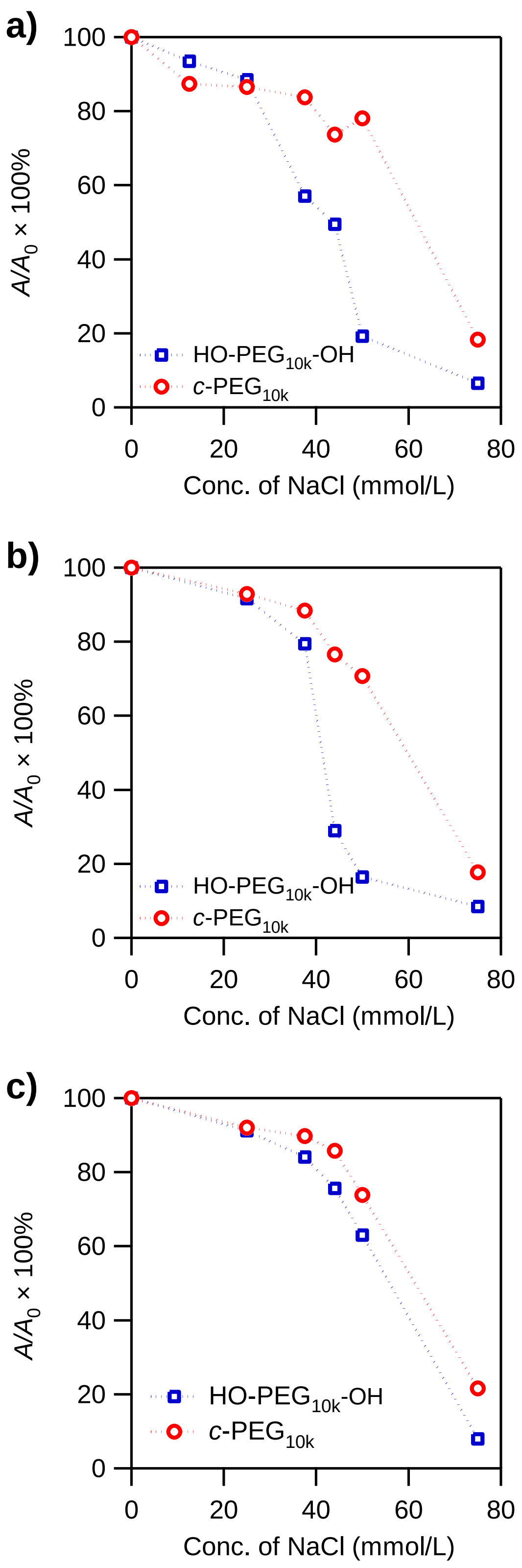


**Figure S18.** Plots of relative UV–Vis absorption intensity ($A/A_0 \times 100\%$) at $\lambda_{max}$ versus the NaCl concentration for the AgNPs prepared in the presence of HO–PEG$_{10k}$–OH (blue) and *c*-PEG$_{10k}$ (red) at (a) $\omega = 2.2$, (b) $\omega = 11$, and (c) $\omega = 44$.
